# CD1d deficiency inhibits the development of abdominal aortic aneurysms in LDL receptor deficient mice

**DOI:** 10.1371/journal.pone.0190962

**Published:** 2018-01-18

**Authors:** Gijs H. M. van Puijvelde, Amanda C. Foks, Rosemarie E. van Bochove, Ilze Bot, Kim L. L. Habets, Saskia C. de Jager, Mariëtte N. D. ter Borg, Puck van Osch, Louis Boon, Mariska Vos, Vivian de Waard, Johan Kuiper

**Affiliations:** 1 Division of Biopharmaceutics, Leiden Academic Centre for Drug Research, Leiden University, Leiden, The Netherlands; 2 Bioceros BV, Utrecht, The Netherlands; 3 Department of Medical Biochemistry, Academic Medical Center, University of Amsterdam, Amsterdam, The Netherlands; Max Delbruck Centrum fur Molekulare Medizin Berlin Buch, GERMANY

## Abstract

An abdominal aortic aneurysm (AAA) is a dilatation of the abdominal aorta leading to serious complications and mostly to death. AAA development is associated with an accumulation of inflammatory cells in the aorta including NKT cells. An important factor in promoting the recruitment of these inflammatory cells into tissues and thereby contributing to the development of AAA is angiotensin II (Ang II). We demonstrate that a deficiency in CD1d dependent NKT cells under hyperlipidemic conditions (LDLr^-/-^CD1d^-/-^ mice) results in a strong decline in the severity of angiotensin II induced aneurysm formation when compared with LDLr^-/-^ mice. In addition, we show that Ang II amplifies the activation of NKT cells both *in vivo* and *in vitro*. We also provide evidence that type I NKT cells contribute to AAA development by inducing the expression of matrix degrading enzymes in vSMCs and macrophages, and by cytokine dependently decreasing vSMC viability. Altogether, these data prove that CD1d-dependent NKT cells contribute to AAA development in the Ang II-mediated aneurysm model by enhancing aortic degradation, establishing that therapeutic applications which target NKT cells can be a successful way to prevent AAA development.

## Introduction

According to the World Health Organization, cardiovascular diseases are the leading cause of death worldwide, responsible for 17.7 million deaths each year, with atherosclerosis, a chronic inflammation of the vessel wall, as the major cause. Another common vascular disorder, linked with aging and atherosclerosis, is the development of an abdominal aortic aneurysm (AAA) affecting 2–8% of the elderly people. AAA, a chronic inflammatory disease, is defined as a local permanent dilation of the abdominal part of the aorta transcending 1.5-times the normal aortic diameter.[1, 2] An AAA is often asymptomatic and undiagnosed until rupture of the aorta occurs. Upon rupture the overall mortality is very high (80% to 90%). Although improved imaging techniques such as X-ray, ultrasound and echocardiogram result in an earlier detection of AAA, surgical intervention is currently the only available treatment. Since no pharmacological therapies exist, there is an urgent need for novel therapeutic strategies to inhibit the progression of AAA.

The inflammatory response, a key process in AAA development, contributes to an increased production of elastase and several proteinases (matrix metalloproteinases (MMPs), serine proteinases, cathepsins), which are mainly responsible for the structural loss of vessel wall integrity leading to AAA formation.[3] Locally increased levels of chemokines (MCP-1[4, 5], CCL22[6], CXCL12[7]), growth factors (GCSF, MCSF)[8] and cytokines (TNF-α, IL-6, IL-1β)[8, 9] cause attraction and accumulation of different leukocytes such as monocytes, macrophages, dendritic cells (DCs), NK cells, neutrophils, B and T cells in the aneurysmal vessel wall.[3, 10] In mice that were depleted for CD4^+^ T cells, AAA formation does not occur.[11] However, contradictory results are observed regarding the role of different T cell subsets in AAA. Foxp3 expressing regulatory T cells are found to be protective in AAA formation in mice,[12] while both pro-inflammatory Th1 cells (producing IL-1β, IL-6, TNF-α and IFN-γ) and anti-inflammatory Th2 cells (producing IL-4, IL-5 and IL-10) are linked to the formation of, as well as the protection against AAA[13–19], confirming the highly complex interplay of different immune cells and cytokines in the pathogenesis of AAA.

NKT cells, another subset of T cells expressing an invariant T cell receptor (TCR) and markers characteristic of NK cells (NK1.1), are also present in large numbers in the aneurysmal vessel wall.[20] While T cells are activated via peptide-antigen presentation on MHC molecules, NKT cells are activated via glycolipid presentation on CD1d, an MHC class I-like molecule. These CD1d-dependent NKT cells comprise a heterogeneous population of cells and based upon differences in TCR characteristics, CD1d-dependent NKT cells are mainly subdivided into type I or type II NKT cells. The most prominent population of NKT cells in mice comprise type I NKT cells, also called invariant NKT (iNKT) cells, expressing a limited diversity in TCRs and all recognizing α-galactosylceramide (α-GalCer). Type II NKT cells express a more diverse range of TCRs and do not respond to α-GalCer. Upon TCR activation and depending on the conditions, NKT cells rapidly and simultaneously produce large amounts of both pro-inflammatory (IFN-γ, IL-2, TNF-α) and/or anti-inflammatory cytokines (IL-4, IL-5, IL-10, IL-13). NKT cells are already linked to the development of atherosclerosis[21–24], but whether the presence of NKT cells in the aneurysmal vessel wall is directly associated with AAA development is still unknown. NKT cells present in AAA tissue predominantly produce pro-inflammatory IFN-γ, which may lead to an upregulated expression of Fas and increased FasL-mediated apoptosis of vascular smooth muscle cells (vSMCs).[20] However, later on it was reported that the anti-inflammatory IL-4, produced by the same NKT cells, might be responsible for increased expression of MMPs by SMCs and macrophages, thereby possibly contributing to the development of AAA.[25–27]

In the current study we establish that LDLr^-/-^ mice lacking CD1d-dependent NKT cells demonstrate reduced AAA severity in the most commonly used model to study the development and pathogenesis of AAA, the angiotensin II (Ang II) infusion model. In addition, *in vitro* studies show that type I NKT cells can contribute, in a cytokine dependent way, to AAA development by increasing the expression of matrix degrading enzymes by macrophages and vSMCs, and by decreasing vSMC viability. In conclusion, CD1d-dependent NKT cells may be a therapeutically interesting target to limit AAA progression.

## Materials and methods

### Animals

All animal work was approved by the Leiden University Animal Ethics Committee and the animal experiments were performed conform the guidelines from Directive 2010/63/EU of the European Parliament on the protection of animals used for scientific purposes. Male C57BL/6, CD1d^-/-^ and LDLr^-/-^ mice on a C57BL/6 background were obtained from our in-house breeding facility. LDLr^-/-^CD1d^-/-^ mice were generated by crossing LDLr^-/-^ mice with the CD1d^-/-^ mice. The offspring was intercrossed to produce mice with a homozygous deletion in both LDLr and CD1d. All mice were kept under standard laboratory conditions (conventional open cages, aspen bedding) in groups of 2–4 mice per cage and were fed a regular chow diet or a ‘Western-type’ diet (WTD) containing 0.25% cholesterol and 15% cocoa butter (Special Diet Services, Witham, Essex, UK). All mice used in experiments were 12–14 weeks of age and of average weight. Diet and water were administered *ad libitum*. At sacrifice, mice were anesthetized by a subcutaneous injection (120 μl) of a cocktail containing ketamine (40 mg/ml), atropine (50 μg/ml) and sedazine (6.25 mg/ml). Subsequently, the mice were euthanized and exsanguinated by femoral artery transection followed by perfusion with PBS through the left cardiac ventricle.

### Media and reagents

NIH/3T3 cells constitutively producing granulocyte-macrophage colony-stimulating factor (GM-CSF)[28] (kindly provided by L. van Duijvenvoorde, LUMC, Leiden, The Netherlands), and bone marrow derived macrophages and dendritic cells (DCs) were cultured in Iscove’s Modified Dulbecco’s Medium (IMDM) containing high glucose, sodium pyruvate, additional amino acids and HEPES (Cambrex, Belgium) supplemented with 8% FCS, 100 U/ml penicillin/streptomycin (Pen/Strep, PAA, Germany), 2 mM Glutamax (Invitrogen, The Netherlands) and 20 μM β-mercaptoethanol (Sigma Aldrich, The Netherlands). NKT hybridoma cells[29] (DN32.D3, kindly provided by R. Raatgeep, Erasmus MC, Rotterdam, The Netherlands) were cultured in DMEM with Glutamax (Cambrex, Belgium) supplemented with 2% FCS, 100 U/ml Pen/Strep, and 1% non-essential amino acids (NEAA) (PAA, Germany). RPMI-1640 (Cambrex, Belgium) supplemented with 10% FCS, 100 U/ml Pen/Strep, 2 mM L-glutamine and 20 μM β-mercaptoethanol was used for spleen cell cultures. Vascular smooth muscle cells (vSMCs), originally isolated from aortas of male C57Bl/6 mice as described before[30], were thawed and cultured in DMEM with 10% FCS, 100 U/ml Pen/Strep and 2 mM L-glutamine and bone marrow derived macrophages were cultured in RPMI with 20% FCS, 100 U/ml Pen/Strep, 2 mM L-glutamine, 1% NEAA and 1% sodium pyruvate (PAA, Germany). All cells were cultured at 37°C and 5% CO_2_.

### AAA induction

AAA was induced by using an Ang II infusion model.[31] In this model, osmotic minipumps (Model 2004, Alzet, DURECT Corporation, Cupertino, USA) were filled with Ang II (Sigma Aldrich, The Netherlands) resulting in a release rate of 1.44 mg Ang II/kg/day. The osmotic pumps were subcutaneously implanted in age-matched LDLr^-/-^ (n = 12) and LDLr^-/-^CD1d^-/-^ mice (n = 11) after anesthetizing the mice with isoflurane. Based upon the results of the study by Liu et al., the mice were put on a WTD one week prior to implementation.[32] Due to the infusion with Ang II and the subsequent development of an aneurysm, sudden death can occur because of a rupture of the aorta. The health of the mice was monitored twice per day during the experiment. Our human endpoint criteria included weight changes (measured once per day), abnormal behavior, changes in the mobility and ruffled fur. None of the mice reached these criteria during the study.

### AAA classification and quantification

Four weeks after placement of the osmotic pumps mice were anesthetized, euthanized and exsanguinated. Subsequently, the mice were perfused through the left cardiac ventricle with PBS for 15 min. The entire aorta is harvested and photographed. Subsequently all the samples were blinded by a colleague not involved in the project and the extent of dilatation of the aorta was determined by measuring the increase in the maximal external diameter of the abdominal (AAA) part, compared with the maximal diameter of the “healthy” part proximal of the aneurysm. The analysis was performed twice by two co-authors. As described by others, a diameter of >150% compared with the healthy situation is considered to be an aneurysm of type I, >200% as a type II, multiple aneurysms and/or dissections as type III and when a mouse died due to aortic rupture it is considered as a type IV aneurysm.[33–35] According to this system, aneurysms varying from type 0 to type IV were assigned a score from 0 to 4 points in order to get a mean score per group of mice.

### Atherosclerotic lesion quantification

After sacrifice, the hearts with aortic root were removed and 10 **μ**m cryosections of the aortic root were made on a Leica CM 3050S Cryostat (Leica Instruments, UK). The sections were stained with Oil-red-O and hematoxylin, and plaque size was measured on blinded slides using a Leica DM-RE microscope and LeicaQwin software (Leica Imaging Systems, UK).

### Cholesterol assay

To determine the cholesterol levels in serum of the mice, blood was collected at different time points during the experiment by tail vein bleeding. Total cholesterol levels were quantified spectrophotometrically using an enzymatic procedure (Roche Diagnostics, Germany). Precipath standardized serum (Boehringer, Germany) was used as an internal standard.

### Immunohistochemistry

After visual inspection, a healthy part and an AAA-affected part of the aorta were embedded in paraffin, sectioned (7 **μm**) and mounted on glass slides (Superfrost-Plus, VWR, The Netherlands). Subsequently, the slides were stained with hematoxylin/eosin. In addition, to detect collagen and MMP-9 the slides were stained with a Masson’s Trichrome staining and an anti-MMP-9 antibody respectively. All images were analyzed using a Leica DM-RE microscope (Leica Imaging Systems, UK).

### Flow cytometric analysis

To determine the effects of Ang II infusion on NKT cell numbers and activation *in vivo*, osmotic minipumps filled with Ang II were implanted in LDLr^-/-^ mice, which were fed a WTD for one week. Two weeks after pump implantation, the mice were sacrificed and perfused as described. Blood was collected, and livers and spleens were dissected and mashed through a 70 μm cell strainer. Erythrocytes were eliminated by incubating the cells with erythrocyte lysis buffer (0.15 M NH_4_Cl, 10 mM NaHCO_3_, 0.1 mM EDTA, pH 7.3). Non-parenchymal cells from the liver were separated from parenchymal cells by centrifugation at low speed. The non-parenchymal cells were put on a Lympholyte gradient (Cedarlane, Ontario, Canada) to isolate liver lymphocytes. Single cell suspensions of liver and spleen were subsequently stained with APC-conjugated α-GalCer/CD1d tetramer (1:800) provided by the NIH tetramer core facility (Atlanta, GA) and PE-conjugated anti-CD25 (0.2 μg/sample) mAb (eBioscience, Belgium) for 30 min. Cells were analyzed by flow cytometry on a FACS Canto II (Becton Dickinson, CA). All data were analyzed with FACS Diva and FlowJo software.

### *In vitro* NKT cell activation

To determine the effect of Ang II on NKT cell activation, bone marrow cells were isolated from the tibia and femurs of LDLr^-/-^ and LDLr^-/-^CD1d^-/-^ mice after euthanization as described above. Cells were cultured for 10 days in IMDM in the presence of GM-CSF. After 10 days, the resulting antigen-presenting cells (APCs) including both macrophages and dendritic cells (DCs) were pulsed with or without α-GalCer (30 ng/ml) and with or without Ang II (100 ng/ml) added to the culture medium. After 4h incubation, the APCs were washed twice. Subsequently, the APCs were co-cultured with NKT hybridoma cells in a 1:5 ratio and after 24h the IL-2 concentration in the supernatant was determined by ELISA according to the manufacturer’s protocol (eBioscience, Austria).

### Real-time PCR assays

To determine effects of NKT cell activation on the expression of proteinases by vSMCs and macrophages, splenocytes from LDLr^-/-^ mice were cultured in a 96-wells plate (2x10^5^ per well) and exposed to the NKT cell specific ligands α-GalCer or OCH (100 ng/ml; Enzo Life Sciences, The Netherlands). After two days the supernatant of the splenocytes was added to bone marrow-derived macrophages and vSMCs, which were then cultured in a 6-wells plate (1.8*10^6^ and 2.5x10^5^ cells per well respectively) in five-fold per condition for three days. Subsequently, mRNA was extracted from the macrophages and vSMCs, using the guanidium isothiocyanate (GTC) method, and reverse transcribed (RevertAid M-MulV reverse transcriptase). Quantitative gene expression analysis for MMP-9, MMP-12, and Cathepsin S, L and K was performed on an ABI PRISM 7700 sequence detector (Applied Biosystems, CA) using SYBR green technology. Acidic ribosomal phosphoprotein PO (36B4), Hypoxanthinephophoribosyl-transferase (HPRT) and ribosomal protein S13 (RPS13) were used as the endogenous reference genes. The primer pairs used are shown in Table 1.

**Table 1:** Primer pairs used for quantitative gene expression analysis.

	forward	reverse
MMP9	5'-CTGGCGTGTGAGTTTCCAAAAT-3'	5'- TGCACGGTTGAAGCAAAGAA-3'
MMP12	5'-CCTGGGCTTCTCTGCATCTGT-3'	5'-CGACGGAACAGGGGGTCATATT-3'
Cathepsin S	5'-GCCAGCCATTCCTCCTTCTTCT-3	5'-TGCCATCAAGAGTCCCATAGCC-3'
Cathepsin K	5'-GGGAACGAGAAAGCCCTGAAGA-3'	5'-ACACTGCATGGTTCACATTATCACG-3'
Cathepsin L	5'-TAGCAGCAAGAACCTCGACCAT-3'	5'-CCATACCCCATTCACTTCCCCA-3'
36B4	5'-GGACCCGAGAAGACCTCCTT-3'	5'-GCACATCACTCAGAATTTCAATGG-3'
HPRT	5'-TTGCTCGAGATGTCATGAAGGA-3'	5'-AGCAGGTCAGCAAAGAACTTATAG-3'
RPS13	5'-TGCTCCCACCTAATTGGAAA-3'	5'-CTTGTGCACACAACAGCATTT-3'

### MTT assay

To investigate the effects of NKT cell specific cytokines on the vSMC viability, supernatant of the splenocytes cultured with α-GalCer or OCH (50, 100 or 200 ng/ml) was again added to vSMC cultures for three days. Viability was assessed by the amount of MTT [3-(4,5-dimethylthiazole-2-yl)-2,5-diphenyltetrazolium bromide] staining (Sigma Aldrich, The Netherlands). Cells were treated with MTT solution (0.5 mg/ml) for 1h and optical density was measured using a spectrophotometer at 550nm. In addition, blocking antibodies against IFN-γ, IL4 or IL-10 (1, 5, 10 and 20 μg/ml, provided by Louis Boon) were added to the supernatant of the splenocytes 30 min before culturing of the vSMCs with this supernatant. An increase in vSMC death was assessed by reduction in MTT staining.

### Statistical analysis

All data are expressed as mean ± SEM. An unpaired two-tailed student’s T-test was used to compare normally distributed data between two groups of animals. A one-way ANOVA with Dunnett’s multiple comparison post-test was performed for multiple comparisons on the same set of data. The Mantel-Cox test was performed to compare the survival distribution between LDLr^-/-^ and LDLr^-/-^CD1d^-/-^ mice. Probability values of <0.05 are considered significant. Data were analysed using GraphPad Prism software (GraphPad Software, La Jolla, CA, USA).

## Results

### Reduced AAA formation in mice lacking CD1d-dependent NKT cells

**Fig 1:**
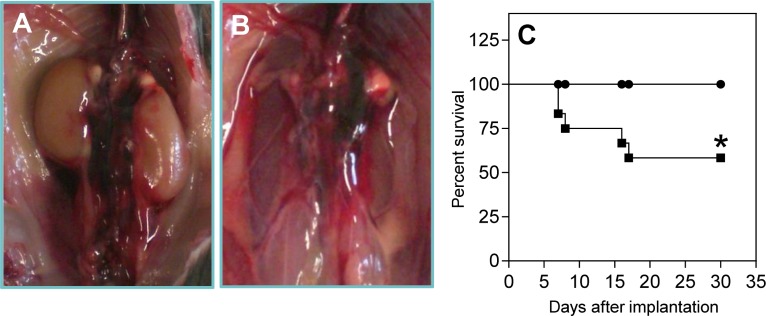
Survival curve of angiotensin II treated LDLr^-/-^ and LDLr^-/-^CD1d^-/-^ mice. Osmotic pumps filled with Ang II were implanted in LDLr^-/-^ (■, n = 12) and LDLr^-/-^CD1d^-/-^ (●, n = 11) which were fed a Western-type of diet for 1 week. After implantation, LDLr^-/-^ mice started to die due to rupture of the abdominal aorta (A and B). Percent survival per group is depicted (C). Statistical analysis was performed using the Mantel-Cox test. *P<0.05.

**Fig 2:**
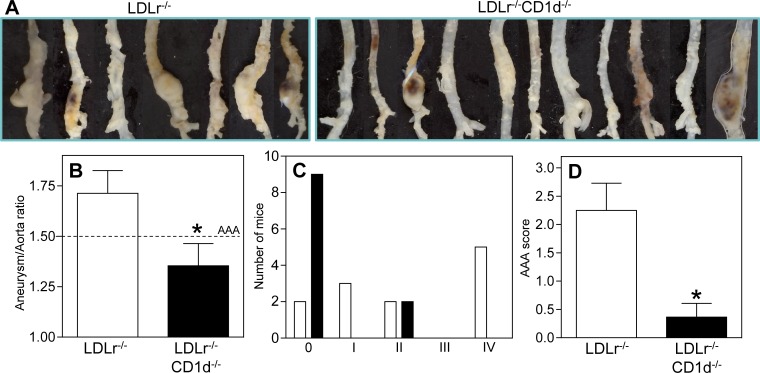
Reduced aneurysm formation in LDLr^-/-^CD1d^-/-^ mice. Osmotic pumps filled with Ang II were implanted in LDLr^-/-^ (n = 12) and LDLr^-/-^CD1d^-/-^ (n = 11) mice which were fed a Western type of diet for 1 week. Aneurysm formation in the surviving LDLr^-/-^ mice (n = 7) and LDLr^-/-^CD1d^-/-^ mice (n = 11) was determined by dissecting the aorta (A) and measuring the ratio between the maximal diameter of the abdominal AAA-affected part and the maximal diameter of the “healthy” thoracic part of the aorta (B). Scoring of the severity of the aneurysms was performed using the visual determination method as described by Daugherty et al., 2011 (C, D). All values are mean±SEM and statistical analysis was performed using the unpaired two-tailed student’s T-test. *P<0.05.

To study the effect of NKT cells on AAA formation, the Ang II-infusion model was used in mice with or without a deficiency in CD1d on an LDLr^-/-^ background. The incidence of AAA and aneurysm severity in both groups of mice was compared. During the experiment, 5 out of 12 LDLr^-/-^ mice died due to an aortic rupture (representative pictures, Fig 1A and 1B), while none of the 11 LDLr^-/-^CD1d^-/-^ mice died (Fig 1C, *P*<0.05). Four weeks after the start of the Ang II treatment, the surviving mice were sacrificed and the aortas were isolated and analyzed (Fig 2A). The diameter of the healthy part of both the thoracic (just proximal of the aortic arch) and abdominal aorta (just above the aortic bifurcation) did not differ between both LDLr^-/-^ and LDLr^-/-^CD1d^-/-^ mice (S1 Fig). The dilatation of the aorta was determined by measuring the maximal diameter of the AAA-affected part and the maximal diameter of the “healthy” part just proximal of the aneurysm. This ratio is 49% lower in LDLr^-/-^CD1d^-/-^ mice (1.35±0.14) when compared with LDLr^-/-^ mice (1.71±0.11, Fig 2B, *P*<0.05), while a ratio of 1 is the physiological situation. Classification of the aneurysms using the visual morphological quantification system for Ang II induced aneurysms in mice,[34] showed a clear difference between both groups. Specifically, 9 out of 11 LDLr^-/-^CD1d^-/-^ mice showed no development of AAA while only 2 out of 12 LDLr^-/-^ mice did not develop an AAA (Fig 2C). After scoring the aneurysms (Type 0 to IV) an 84% reduction in aneurysm severity was observed in LDLr^-/-^CD1d^-/-^ mice (2.25±0.63 vs. 0.36±0.24, Fig 2D, *P*<0.05). Immunohistochemical staining of the AAA tissue showed clear breaks in the elastic lamina and an increased number of MMP-9 expressing cells (S2 Fig). Total cholesterol levels did not differ between LDLr^-/-^ and LDLr^-/-^CD1d^-/-^ mice before (441±20 vs. 424±17 mg/dl, respectively) and after Ang II perfusion (1416±104 vs. 1460±83 mg/dl, respectively, S3 Fig). LDLr^-/-^CD1d^-/-^ mice had a not significant lower body weight at the beginning of the experiment when compared with the age-matched LDLr^-/-^ mice and during the experiment there was no significant difference in weight gain between both groups of mice (S3 Fig). Additionally, a 31% decrease in atherosclerotic lesion size was detected in the aortic root of LDLr^-/-^CD1d^-/-^ mice compared with LDLr^-/-^ mice, although this decrease was not significant (59085±6531 μm^2^ vs. 85385±19987 μm^2^, S3 Fig, *P* = 0.157).

### Ang II stimulates NKT cell activation *in vivo* and *in vitro*

**Fig 3:**
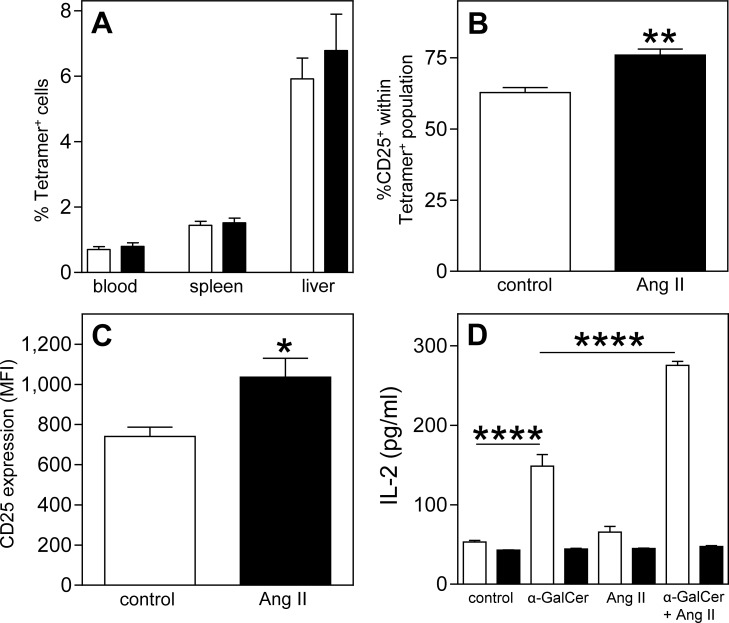
Increased NKT cell activity upon angiotensin II treatment. Osmotic pumps filled with PBS (n = 5) or Ang II (n = 5) were implanted in LDLr^-/-^ mice fed a Western type diet for 1 week. Two weeks after pump placement, the mice were sacrificed and the percentage of NKT (Tetramer^+^) cells in spleen and liver (A, white bars represent PBS treated mice, black bars Ang II treated mice) and the activation status of splenic NKT cells (B and C) were determined by FACS analysis. To confirm these effects, antigen-presenting cells (APCs) isolated from bone marrow of LDLr^-/-^ (white bars) and LDLr^-/-^CD1d^-/-^ mice (black bars) were incubated with α-GalCer, AngII or a combination of both. Four hours after incubation, the APCs were co-cultured with DN32.D3 hybridoma cells. After 24 hours, the IL-2 concentration in the supernatant was determined (D). All values are mean±SEM and statistical analysis was performed using the unpaired two-tailed student’s T-test (A-C) or one-way ANOVA (D) *P<0.05, **P<0.01, ****P<0.0001.

To investigate whether Ang II influences type I NKT cells *in vivo*, a FACS analysis was performed on blood, liver and spleen of LDLr^-/-^ mice after 2 weeks of Ang II infusion. FACS analysis showed that Ang II did not affect the percentages of α-GalCer/CD1d-tetramer^+^ NKT cells in the circulation, spleen and liver (Fig 3A). However, Ang II induced an activation of these NKT cells. The percentage of splenic type I NKT cells expressing CD25 increased with 21% (76.0±2.1% vs. 62.8±1.8%, Fig 3B, *P*<0.01) while the expression level of the activation marker CD25 on type I NKT cells increased with 40% (MFI of 1036±94 vs. 741±46, Fig 3C and S3 Fig, *P*<0.05). Additionally, circulating IFN-γ and IL-4 levels, both key cytokines produced by NKT cells, were measured in serum of saline and Ang II treated mice but both cytokine levels were below the detection limit in both groups of mice. To confirm the enhanced NKT cell activation upon exposure to Ang II, DN32.D3 NKT hybridoma cells were co-cultured with pre-treated APCs obtained from bone marrow of LDLr^-/-^ or LDLr^-/-^CD1d^-/-^ mice. A significant increase in the production of IL-2 by NKT cells was observed after exposure to α-GalCer pulsed APCs (148.7±14.5 pg/ml) when compared to unpulsed APCs (53.0±2.2 pg/ml, *P*<0.0001). This effect was significantly amplified by the addition of Ang II (275.4±5.0 pg/ml, Fig 3D, *P*<0.0001) while Ang II alone had no effect (65.6±7.1 pg/ml). These effects were absent when α-GalCer and Ang II pulsed LDLr^-/-^CD1d^-/-^ APCs were co-cultured with DN32.D3 cells.

### Increased expression of proteases after NKT cell activation

**Fig 4:**
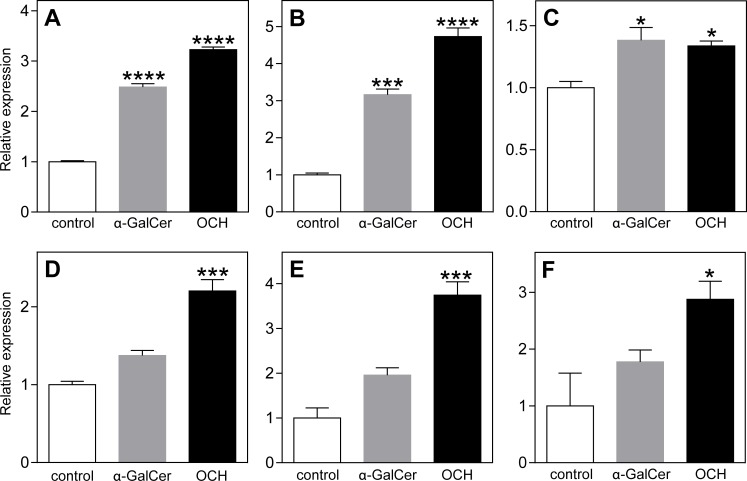
Increased expression of matrix degrading molecules by vSMCs and macrophages after type I NKT cell activation. Splenocytes of LDLr^-/-^ mice were incubated with or without type I NKT cell specific ligands α-GalCer and OCH for 48 hours. Subsequently, supernatant of the splenocytes was added to vSMCs (A-C) or macrophages (D-F) for 72 hours after which the mRNA expression of the matrix degrading molecules Cathepsin S (A and D), MMP-12 (B and E), Cathepsin K (C) and Cathepsin L (F) was determined. All values are mean±SEM and statistical analysis was performed using one-way ANOVA. *P<0.05, ***P<0.001, ****P<0.0001.

To investigate how type I NKT cells could contribute to the development of AAA, splenocytes of LDLr^-/-^ mice were cultured for 48h in the presence of the type I NKT cell specific ligands α-GalCer or OCH. α-GalCer is known to induce a mixed Th1/Th2 (especially IFN-γ) cytokine profile[36], while OCH more specifically induces NKT cells to produce Th2 cytokines (IL-4 and IL-10).[37, 38] Subsequently, vSMCs and macrophages were exposed to supernatant of these splenocytes for 3 days, after which the expression of several matrix degrading proteinases was determined. Incubation of vSMCs with conditioned medium of the α-GalCer- or OCH-treated splenocytes caused a significant increase in mRNA expression of Cathepsin S, MMP-12 and Cathepsin K (Fig 4A–4C, respectively). Incubation of bone marrow-derived macrophages with conditioned medium of OCH-treated splenocytes increased the expression of Cathepsin S, MMP-12, and Cathepsin L (Fig 4D–4F, respectively). These significant effects on macrophages were not observed after the addition of conditioned medium from α-GalCer-treated splenocytes, indicating that especially Th2 cytokines (produced after OCH) induce the expression of proteases by macrophages.

### Increased vSMC apoptosis after NKT cell activation

**Fig 5:**
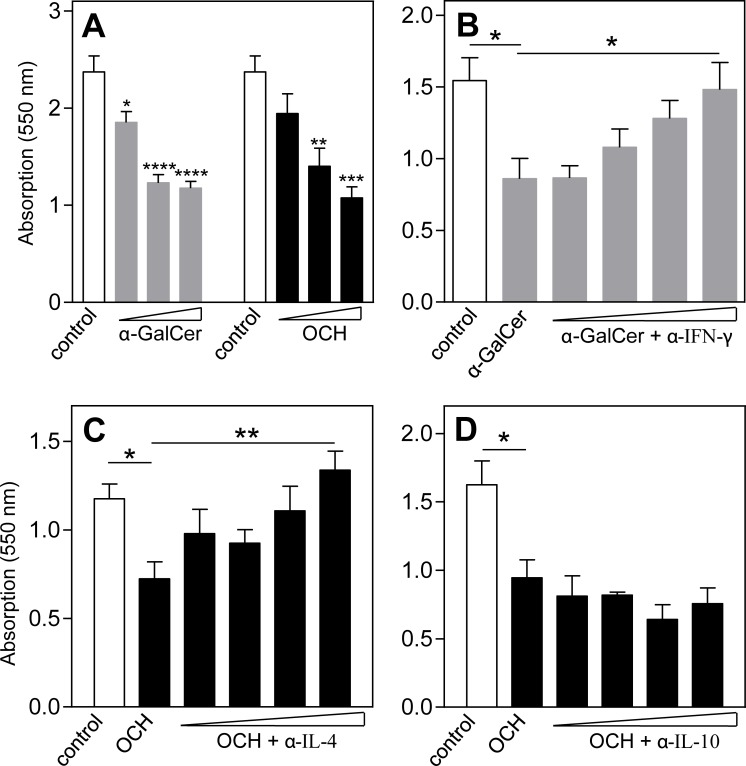
Cytokine dependent decrease in vSMC viability after type I NKT cell activation. Splenocytes were cultured in presence of type I NKT cell specific ligands α-GalCer or OCH (50, 100 and 200 ng/ml) for 48 hours. Subsequently, supernatant of the splenocytes was added to vSMCs for 72 hours and viability of the vSMCs was assessed by an MTT assay (A). In addition, supernatant of the α-GalCer (200 ng/ml) stimulated splenocytes was pre-incubated with α-IFN-γ antibodies (B) and supernatant of OCH (200 ng/ml) stimulated splenocytes with α-IL4 (C) and α-IL10 (D) antibodies prior to addition of the supernatant to vSMCs. All values are mean±SEM and statistical analysis was performed using one-way ANOVA. *P<0.05, **P<0.01, ***P<0.001, ****P<0.0001.

To further study the effect of type I NKT cell activation on the development of AAA, splenocytes were cultured with different concentrations of α-GalCer or OCH for 48h. Subsequently, vSMCs were exposed to conditioned medium from these splenocytes and after 72h, the viability of the vSMCs was assessed using an MTT assay. Addition of conditioned medium from α-GalCer- and OCH-treated splenocytes decreased the viability of vSMCs in a dose-dependent manner (Fig 5A). This decrease in viability could be counteracted dose-dependently by adding increasing concentrations of IFN-γ (Fig 5B) and IL-4 (Fig 5C) blocking antibodies, respectively. The addition of an IL-10 blocking antibody did not counteract the OCH-induced decrease in vSMC viability (Fig 5D).

## Discussion

During the development of aneurysms, both innate and adaptive inflammatory processes contribute to the progression of disease. Inflammatory cells such as monocytes/macrophages, neutrophils, T cells (different subsets including Th1, Th2 and NKT cells), B cells, mast cells and NK cells infiltrate the vessel wall and locally secrete cytokines, chemokines and other inflammatory factors resulting in the progression of AAA.[3, 10] CD4^+^ T cells dominate these infiltrates,[14] but it is still unclear which T cell subclass is responsible for the pathology of AAA. Both pro-inflammatory Th1 cells secreting IFN-γ and thereby increasing the MMP-2 and MMP-9 expression[11, 14] and anti-inflammatory Th2 cells secreting IL-4 and concomitantly increasing the expression of MMP-9 and MMP-12 seem to be involved in AAA development.[18, 39] However, Th1 cells are also linked to protection against the formation of AAA, since blocking of IFN-γ signalling pathways induced AAA formation in allografted aortas.[18]

In this study we show for the first time that a deficiency in CD1d-dependent NKT cells, a distinct subset of lipid sensing T cells, leads to a substantial reduction in the incidence and severity of aneurysms in the Ang II infusion model for AAA in mice. No aortic ruptures were detected in the LDLr^-/-^CD1d^-/-^ mice, which lack functional type I and type II NKT cells, while this is a regular phenomenon after Ang II infusion in hyperlipidemic apoE^-/-^ and LDLr^-/-^ mice.[40, 41] This proved that CD1d-dependent NKT cells contribute to the progression of the disease.

It is known that Ang II acts systemically on several cell types such as endothelial cells, vascular smooth muscle cells, dendritic cells, monocytes and lymphocytes, in both mice (Ang II infusion model) and men.[42] In the present study we observed an increased activity of NKT cells upon the administration of Ang II, both *in vitro* and *in vivo*. Incubation of APCs with Ang II enhanced the α-GalCer-induced activation of NKT cells, suggesting an indirect effect of Ang II on type I NKT cell activation. This might be due to activation of receptors for Ang II such as the AT1 receptor, leading to augmented differentiation and function of DCs.[43, 44] In vivo we observed an increased expression of the activation marker CD25 on NKT cells after treatment with Ang II. Concomitantly, you would expect increased levels of NKT cell-derived cytokines however IFN-γ and IL-4 levels in the serum of Ang II treated mice remained undetectable. Future research should provide evidence whether this Ang II-induced improvement of the activation of NKT cells is due to the augmented function of APCs present in the spleen or whether it is caused by the endogenous renin-angiotensin system of NKT cells[45] modulating their function as is already shown for regular T cells.[46].

In a previous study it was speculated that NKT cells contribute to the breakdown of the vessel wall by their specific interaction with vSMCs.[20] Especially NKT cells with a pro-inflammatory phenotype, mainly producing IFN-γ, induce Fas/FasL-mediated apoptosis of vSMCs. However, the same authors also speculated that the anti-inflammatory IL-4, produced by the same NKT cells, is responsible for an increased expression of MMPs by vSMCs and macrophages, thereby contributing to the AAA development.[25] We now show that the activation of type I NKT cells by the specific ligands α-GalCer and OCH results in a significant upregulation in the expression of different matrix degrading proteinases by both vSMCs and macrophages. While activation by α-GalCer, preferentially inducing the production of the Th1-cytokine IFN-γ and equivalent to the pro-inflammatory conditions in hyperlipidemic mice and men, mainly affects vSMCs, activation by OCH, inducing the production of the Th2-cytokine IL-4, affects both vSMCs and macrophages. Our results are in line with several other studies on the contribution of different cytokines to the expression of matrix degrading enzymes, and subsequently to the progression of AAA. IL-4 enhances the proteolytic capacity of macrophages by increasing the expression of Cathepsin S and L[47], and alternative activation of macrophages by IL-4 also induced the expression of MMP-12.[48] Upon IL-4 treatment, vSMCs start expressing several proteases such as MMP-1[26], while incubation of vSMCs with IFN-γ also leads to an increased expression of several proteases such as Cathepsin S.[49]

In addition, we found that the activation of type I NKT cells leads to reduced vSMC viability. We showed that this effect can either be induced by IFN-γ, as described previously[20, 50], but also by IL-4 since blocking of IFN-γ and IL-4 reduced the apoptosis of vSMCs induced by α-GalCer or OCH-activated NKT cells, respectively. In conclusion, both Th1 and Th2 cytokines produced by type I NKT cells may be responsible for the contribution of type I NKT cells to AAA development. Since circulating levels of these NKT cell-derived cytokines remained undetectable after Ang II infusion, we speculate that, although the increased activation status of NKT cells upon Ang II infusion seems to be systemic, especially locally produced cytokines may be responsible for weakening of the vessel wall structure leading to AAA development. Although we showed increased expression of MMP-9 in the vessel wall of mice with AAA, our study lacks data on the activity of MMPs and cathepsins in both the LDLr^-/-^ and LDLr^-/-^CD1d^-/-^ mice after Ang II infusion.

Another limitation to our study is the lack of data on blood pressure. In addition to the involvement of Ang II in key events of inflammatory processes contributing to AAA development, Ang II also induces AAA development by elevating the blood pressure. It would have been interesting to determine whether Ang II infusion differentially affects blood pressure in LDLr^-/-^ and LDLr^-/-^CD1d^-/-^ mice, but we did not measure this in both Ang II infusion experiments.

Correlation studies showed that there is a positive association between coronary heart disease and peripheral atherosclerosis with AAA development.[51] It is however still unclear whether atherosclerosis is directly leading to AAA formation or whether atherosclerosis and AAA develop in parallel.[52] In our present study, and as shown before, hyperlipidemic mice which are deficient in functional NKT cells (LDLr^-/-^CD1d^-/-^ mice), have smaller atherosclerotic lesions compared with LDLr^-/-^ mice, especially during the initial phase of the disease.[22, 23] Therefore, in addition to a direct role of NKT cells in the AAA development, NKT cells might also contribute to AAA formation by their pro-atherogenic function although the correlation between atherosclerosis and the severity of AAA in our study is not quite strong (R^2^ = 0.167, *P* = 0.09).

Currently, no pharmacological therapies to reduce the progression of AAA exist, and the treatment of patients is limited to surgical intervention. Although less invasive surgical techniques have evolved recently, there is an urgent need for novel therapeutic strategies to inhibit AAA progression. Our results reveal that targeting CD1d-dependent NKT cells may be a successful therapeutic approach to prevent AAA development. At the moment, two possible strategies to inhibit the activation of NKT cells in vivo are known and will be tested in the context of AAA in the future. One by blocking the interaction between the TCR of NKT cells and CD1d with anti-CD1d antibodies,[53, 54] and one by preventing the presentation of ligands on CD1d with the lipid antagonist DPPE-PEG[55, 56].

## Supporting information

S1 Fig Aortic diameter of LDLr^-/-^ and LDLr^-/-^CD1d^-/-^ mice. After sacrifice of the mice, the diameter of the healthy part of both the thoracic (just proximal of the aortic arch) and abdominal aorta (just above the aortic bifurcation) of LDLr^-/-^ and LDLr^-/-^CD1d^-/-^ mice were determined.
(EPS)

S2 Fig Reduced aneurysm formation in LDLr^-/-^CD1d^-/-^ mice. Paraffin sections of the aorta of mostly unaffected LDLr^-/-^CD1d^-/-^ (A, B and E) and mostly affected LDLr^-/-^ mice (C, D, F-H) were stained with hematoxylin/eosin (A and C), trichrome (B and D) and an α-MMP-9 antibody (E-G). A negative control for the MMP-9 staining is shown in H. Red arrows in C and D mark breaks in the elastic lamina of the aortic vessel wall of LDLr^-/-^ mice.
(EPS)

S3 Fig Cholesterol levels, weight, atherosclerotic plaque formation and NKT cell activation. Osmotic pumps filled with Ang II were implanted in age-matched LDLr^-/-^ (n = 12) and LDLr^-/-^CD1d^-/-^ (n = 11) which were fed a Western type diet for 5 weeks in total. Cholesterol levels (A) and weight (B) were measured during the experiment (■ represent LDLr^-/-^ mice, ▲ represent LDLr^-/-^CD1d^-/-^ mice) and atherosclerotic lesion development at the aortic root (C) was determined after the experiment. CD25 expression of splenic NKT cells after Ang II infusion was determined by FACS analysis (D, green = isotype control, blue = PBS treated mice, red = Ang II treated mice). All values are mean±SEM and statistical analysis was performed using the unpaired two-tailed student’s T-test.
(EPS)

S1 File Data set. (XLSX)

S2 File ARRIVE guidelines. (PDF)
